# Digital leadership and employee innovative performance: the role of job crafting and person–job fit

**DOI:** 10.3389/fpsyg.2025.1492264

**Published:** 2025-04-30

**Authors:** Yongkang Wang, Jonghyuk Park, Qi Gao

**Affiliations:** ^1^Graduate School, Kangnam University, Yongin, Republic of Korea; ^2^Division of Global Business Administration, Kangnam University, Yongin, Republic of Korea; ^3^Business School, Shandong University of Technology, Shandong, China

**Keywords:** digital leadership, task crafting, cognitive crafting, relational crafting, person-job fit, employee innovative performance

## Abstract

With the development of the digital economy and digital technology, innovation-driven growth has become the key to the digital transformation of various organizations. Employee behavior and digital leadership affect the innovative performance of a company significantly. Using the proactive motivation model, this study constructed a moderated mediation model with job crafting as the mediating variable and person–job fit as the moderating variable. Through statistical analysis of 306 valid questionnaires answered by employees in manufacturing firms, this study determined how digital leadership affects innovative performance by promoting employees to carry out job crafting. The study conducted structure equation modeling to examine the hypotheses. The findings indicate the following: (1) Digital leadership has a positive effect on employee innovative performance. (2) Two of the three job crafting strategies (task crafting and cognitive crafting) mediate the relationship between digital leadership and employee innovation performance. (3) Person–job fit positively moderates the relationship between cognitive crafting and employee innovation performance. (4) Person–job fit positively moderates the indirect effect of digital leadership on employee innovation performance through cognitive crafting.

## 1 Introduction

The current global economy is characterized by digitalisation, and society is entering a new phase dominated by digital productivity. Digitalisation has also become a core driver of business growth (Duan et al., 2019), with enterprises embracing digital transformation. For companies such as Google, Apple, and TikTok, recognizing and exploiting digitalisation opportunities has become second nature. However, for many enterprises, it remains a new concept. In particular, Asian enterprises are grappling with the complexities of modern product ecosystems and manufacturing processes driven by big data analytics, especially datasets generated by digital devices, during their digital transformation journeys (Park et al., 2023). This shift requires manufacturers to reconcile traditional operational frameworks with data-intensive production systems. In the current digital transformation landscape, innovation has become more crucial than ever for organizational sustainability (Curzi et al., 2019). Companies need to determine how innovation contributes to organizational growth and, in turn, enhances organizational performance. The innovation performance of an enterprise depends on the creative ideas and actions of individual employees (Yuan and Woodman, 2010). As employees are at the core of enterprise innovation, improving their innovation performance will directly drive enterprise innovation. In this context, studying employee innovation performance is particularly important, as it is a complex issue influenced by various factors.

Leadership is an important factor influencing employee innovation and is critical to the digital transformation of organizations (Abbu et al., 2022). Leaders must possess digital competencies to inspire employee innovation, thereby enhancing their overall performance (Abbas et al., 2024). In this context, digital leadership stands out as a key factor. A survey conducted by the World Economic Forum (2020) involving 800 global enterprises revealed that organizations with higher digital maturity and well-defined digital strategies led by competent leaders experienced an average revenue growth of 12%, whereas companies lacking digital leadership saw only a 3% growth. Digital leadership is defined as a set of competencies and behavioral processes through which leaders leverage digital technologies to drive changes in attitudes, emotions, cognition, behaviors and performance, thereby creating value and enabling organizational digital transformation (Lin, 2024). The primary objective of digital leadership is enhancing organizational value and improving business performance (Benitez et al., 2022), while also facilitating digital transformation through its influence on organizational structures (Artüz and Bayraktar, 2021; Skopak and Hadzaihmetovic, 2022). Under the guidance of digital leaders, employees can focus on developing novel ideas and actively explore innovative solutions that align with organizational objectives (Erhan et al., 2022). Although previous studies have suggested that digital leadership can enhance and encourage innovative work behaviors and capabilities in employees (Hadi et al., 2024; Sagbas et al., 2023; Sasmoko et al., 2019), the mechanisms through which digital leadership influences innovation performance remain unclear (Mihardjo et al., 2019). Therefore, this study first examines the impact of digital leadership on employee innovation performance.

In the digital age, the dynamics of the environment and increased awareness of the individual employee mean that companies need to change their perspective and focus on proactive employee behavior. Concomitantly, employees in organizations can redefine and redesign their jobs in response to the challenges of digitalisation to ensure a good employee–job fit in the work environment (Tims et al., 2012). Job crafting is how employees play an active role in proactively changing the physical, cognitive or social characteristics of their work. It is an informal process through which employees redesign their work to align it with their interests and values. As such, job crafting is a proactive behavior initiated by employees from the bottom-up, rather than driven by the management (Grant and Ashford, 2008). Wrzesniewski and Dutton (2001) introduced this idea in their original conceptualisation of job crafting. They proposed three forms of job crafting: task crafting, cognitive crafting, and relational crafting. In the process of making task, relational and cognitive changes to the boundaries of their work, the meaning of work and the identity of the employees change (Slemp and Vella-Brodrick, 2013). Cognitive crafting is crucial in job crafting and is considered the aspect most closely related to the meaning of work and work identity (Zhang and Parker, 2019). Focusing on changes in employee perceptions is important because it can produce important personal outcomes related to wellbeing, which are beneficial to the sustainability of enterprises (Harter et al., 2003; Hodges and Clifton, 2004). The job crafting behavior of employees will further affect their performance and creativity (Tims et al., 2015; Rudolph et al., 2017; Zhu et al., 2022). Therefore, this study examines the mediating role of three forms of job crafting (task, cognitive and relational) in the relationship between digital leadership and employee innovation performance. Furthermore, this study is grounded in the proactive motivation model, which posits that employee proactive behaviors are driven by three motivational states: “Can Do” (self-efficacy), “Reason To” (goal alignment) and “Energized To” (affective commitment) (Parker et al., 2010). Specifically, digital leaders can enhance these employee motivational states by providing digital technology support, clarifying innovation goals, and fostering team collaboration. These efforts, in turn, stimulate employee proactive behaviors and improve their innovation performance. This study offers novel insights into the dynamic relationships among digital leadership, job crafting and employee innovation performance.

Additionally, this study explores the boundary conditions that may moderate the relationship between job crafting and employee innovation performance. Person–environment (P–E) fit theory emphasizes that at the individual level, “a person is defined by their knowledge, skills, abilities and other characteristics, such as personality, values, and interests” (Tepper et al., 2018). The core dimensions of P–E fit theory include person–organization (P–O) fit and person–job (P–J) fit (Kristof-Brown et al., 2005). Historically, P–E fit theory has primarily focused on P–O fit, as role-based actions within organizational structures were considered crucial. However, with the increasing demand for employee innovation, P–J fit has become more important. Further, P–J fit increases job satisfaction and organizational commitment and reduces turnover intentions (Hambleton et al., 2000; Lauver and Kristof-Brown, 2001). Therefore, this study examines the moderating role of P–J fit in the relationship between job crafting and employee innovation performance.

The contribution of this study to existing research is reflected in the following aspects: First, this study used job crafting as the mediating variable to analyze how digital leadership affects employee innovative performance. To this end, the study investigated the mediating effect of job crafting in various dimensions, thereby elucidating the intrinsic influence mechanism of digital leadership on employee innovative performance. Second, this study also examined the moderating effect of P–J fit, i.e., how P–J fit moderates the relationship between job crafting and employee innovation performance.

## 2 Theory

### 2.1 Proactive motivation model

The proactive motivation model, introduced by Parker et al. (2010), explains how individuals cultivate proactive behaviors through a motivated, deliberate and goal-directed process. This process is governed by three key motivational states: “Can Do,” “Reason To,” and “Energized To.” Engaging in proactive behavior often entails setting ambitious goals to challenge the status quo, necessitating a strong belief in one’s ability to succeed (“Can Do”). First, the “Can Do” motivation reflects employees’ confidence in their abilities and their assessment of task feasibility, primarily rooted in self-efficacy and control appraisals (Bandura, 1997; Frese and Fay, 2001). Second, the “Reason To” motivation emphasizes the intrinsic and extrinsic drivers behind employees’ proactive pursuit of specific goals. This motivation is grounded in self-determination theory, intrinsic motivation and integrated motivation (Deci and Ryan, 2000). Finally, the “Energized To” motivation underscores the role of positive emotional states, such as enthusiasm and excitement, in fuelling employees’ proactive behaviors.

The proactive motivation model elucidates how digital leadership shapes employees’ innovation performance via job crafting, highlighting the distinct motivational mechanisms it provides. As a vital organizational resource, digital leaders act as key drivers of motivation by strengthening employees’ “Can Do” motivation (offering technological support and training), stimulating “Reason To” motivation (enhancing autonomy and instilling new values) and fostering “Energized To” motivation (cultivating an innovative environment and facilitating team collaboration) (Sun et al., 2024; Alvarez-Torres and Schiuma, 2022; Hanelt et al., 2021). These three motivational states interact synergistically to drive employees’ proactive behaviors (i.e., job crafting) within digital transformation, ultimately boosting their innovation performance.

### 2.2 Job crafting

The new generation of young knowledge workers make up a larger proportion of the organizational workforce; they are more self-centered than their predecessors, giving more attention not only to pay, but also to work experience and the value of work for themselves and the community; they also aspire to perform meaningful work. They have started to realize that within the prescribed framework of work, they can take the initiative to make some changes to their original work according to their needs, so that the work is more in line with their preferences and strengths, and better aligns with their skills, values, and motivations (Rousseau et al., 2006). Against this background, changing the traditional top-down approach to work and the behavior of employees in redesigning their work based on their initiative and spontaneity has received increasing attention from organizational managers and researchers (Kulik et al., 1987).

The concept of job crafting was first introduced by Wrzesniewski and Dutton (2001), who defined it from a role perspective as employees proactively changing their job boundaries and making substantive or cognitive changes in tasks and relationships to better match their work with their skills, preferences, and values, thereby meeting their individual needs. They analyzed job crafting based on three dimensions. Task remodeling refers to the initiative of an individual to alter the number, scope, and type of tasks at work. Relational remodeling refers to the initiative of an individual to modify the quality and quantity of interpersonal interactions at work. Cognitive remodeling refers to the initiative of individuals to alter their original views and ideas at work (Wrzesniewski and Dutton, 2001).

As job crafting is an employee initiative, it is described as a personalized, bottom-up, and proactive design method, as opposed to the top-down and “one size fits all” work design methods initiated by enterprises (Grant and Parker, 2009; Parker, 2014). Through job crafting, employees can change the task, cognitive, and relational boundaries of their work (Petrou et al., 2018). Employees who change any of these elements change the job design and social context of their work. These behaviors change the meaning of work and the identity of an employee at work (Lyons, 2008). Therefore, this study adopted the definition of job crafting provided by Wrzesniewski and Dutton and examined each of the three dimensions.

Job crafting has important characteristics. First, it is self-focused and designed to benefit the individual for whom job crafting occurs (Tims et al., 2012; Wrzesniewski and Dutton, 2001). Second, it is a self-directed, bottom-up behavior that is an employee-initiated self-transformation (Tims and Bakker, 2010; Wrzesniewski and Dutton, 2001). Third, job crafting behaviors are sustainable and not one-off or temporary changes (Bruning and Campion, 2018; Wrzesniewski and Dutton, 2001). Finally, the purpose of individual work redesign is to achieve a match between the individual and the environment, thereby enhancing work meaning, motivation, wellbeing, and performance (Tims et al., 2013; Wrzesniewski and Dutton, 2001).

## 3 Hypotheses

### 3.1 Digital leadership and employee innovative performance

With the rise in digital technologies and digital economy, most enterprises that compete globally have become digital companies (Abollado and Shehab, 2018; Barchiesi and Colladon, 2021), and traditional processes and business models are changing (Holzmann et al., 2020; Wesseling et al., 2020). The digital age requires new capabilities to create a sense of digital urgency to drive this vision and implement appropriate leadership models (Kohnke, 2017). Digital leadership is defined as a leader’s ability to integrate competencies with digital technologies to generate organizational value (Rudito and Sinaga, 2017). Digital leaders are pivotal in steering organizational digital transformation. They confer competitive advantages to organizations by adopting a strategic perspective and flexibly adapting to diverse leadership styles, including transformational and transactional leadership (Sow and Aborbie, 2018). The leadership style of digital leaders is fast-paced, team-centric, collaborative, cross-functional and highly innovation-focused (Oberer and Erkollar, 2018). Strong digital leaders will possess new skills to help their organization effectively navigate the uncertainty and complexity surrounding them, assisting the business in defining a digital strategy and enhancing business performance (de Araujo et al., 2021).

Employee innovation performance results from innovation in the work of employees and refers to the process in which employees proactively propose, promote and implement new ideas, methods or processes within the organization (Janssen, 2000). Digital technology brings new business models and has a borderless impact on innovation (Zhu, 2015). Digital leaders play an important role in nurturing the creativity of employees and possess creativity and innovative thinking to turn ideas into reality for the enterprise. Digital leaders drive innovation and digitisation in enterprises, where information and knowledge are shared rapidly, so that every employee can access, process and apply them (Wasono and Furinto, 2018). Digital leaders demonstrate adaptability and openness to new ideas and technologies (Bennis, 2013), enabling rapid dissemination of beneficial new concepts generated by employees and expeditious application of new products and ideas.

Digitalization has transformed the working environment and needs of enterprises. Leaders no longer merely assign tasks to subordinates and monitor their completion; they also create space for employees to realize their creative potential through collaboration and continuous learning (Bass and Riggio, 2006). Digital leaders should empower employees to engage in innovative activities and create an innovative atmosphere (Zhu et al., 2022) to promote the implementation of innovative ideas for achieve innovative outcomes, thereby enhancing employee innovative performance. Existing research indicates that digital leadership enhances innovation performance (Benitez et al., 2022), strengthens service innovation capability (Brunner et al., 2023) and promotes open innovation (Fatima and Masood, 2024). Furthermore, digital leadership positively moderates the relationship between digital technology utilization and innovation capability (Borah et al., 2022). Hence, we propose that digital leadership positively influences employee innovation performance.

*H1*: Digital leadership is positively related to employee innovative performance.

### 3.2 Mediating effect of job crafting between digital leadership and employee innovative performance

Enterprises facing a rapidly changing external environment and increasing complexity in internal tasks need more flexibility to manage complex and challenging team tasks. It is unlikely that one person can do this (Edelmann et al., 2020). Therefore, enterprises need digital leaders who encourage employees to participate in the overall process. This form of leadership enables employees to express different ideas (Abbasi et al., 2020). In addition, the increasing number of knowledge-based employees in enterprises is changing the employee hierarchy (Nurhidayati and Zaenuri, 2023). Most of them believe that they can adjust the order, manner or scope of their tasks according to the specifics of their work, rather than just carrying out their duties per the inherent requirements of enterprises (Berg et al., 2010). Job crafting breaks the previous passive management approach, where employees proactively adjust job tasks, cognition and relationships to adapt to the new environment.

As digital leadership and digital technologies change how employees work and the scope of their work (Trenerry et al., 2021), employees need to change their traditional ways of working and the work processes, adjust the definition of digital work boundaries and perform digital work tasks effectively for adapting to digital transformation (Davison and Ou, 2017). Digital leadership inspires employees to explore emerging technologies and embrace innovative problem-solving approaches (Dery et al., 2017). Digital leaders are responsible for providing essential resources, implementing structural changes and guiding employees in adapting to evolving work practices (Trenerry et al., 2021; Chanias et al., 2019; Selimović et al., 2021). In line with the proactive motivation model, digital leadership fosters employees’ self-efficacy and controls appraisals by offering technological support and resources, thereby reinforcing their “Can Do” motivation and bolstering their confidence in job crafting. Employees can achieve task crafting by changing the quantity, type, scope or method of digitized tasks, thereby aligning their interests and motivations more closely with the requirements of their jobs. Digital leaders are adept at using digital resources, such as digital devices and services, to establish relationships among organizational members (Van Wart et al., 2019). Employees can build and maintain quality relationships by increasing the frequency of interactions with others in the workplace. High-quality collegiality promotes the exchange of experiences in using digital technologies to help each other adapt to digital transformation and enhancing employees’ positive emotions, thereby strengthening their “Energized To” motivation and changing employee relations. Digitalisation is not just a technological change, but it is a revolution in cognitive thinking. The attitude of employees toward digital technology affects their use of digital technology and job performance (Khin and Ho, 2019). Digital leaders have a strong willingness to engage in role learning and role sensitisation to improve their digital skills and literacy, articulate knowledge related to digital technologies, develop employee trust in the virtual environment and improve organizational resilience to the digital environment (Larjovuori et al., 2016). Changing employee perceptions and value judgements about the relationship between digital technologies and digital work demands as well as personal preferences can increase positive perceptions of digital technologies, and stimulate intrinsic and integrated motivation, thereby strengthening their “Reason To” motivation (Deci and Ryan, 2000) and fundamentally changing the cognitive attitudes of employees toward digital technologies and digital work demands (Kane et al., 2019).

Employee job crafting can also positively impact employee innovation performance. First, employee involvement in job crafting can stimulate creativity, job satisfaction, positive job identity, work-related wellbeing and job performance (Tims et al., 2012). An increase in work resources, engagement, and satisfaction enhances employees’ intrinsic motivation, encouraging them to engage in innovative behaviors to perform their jobs efficiently (Demerouti et al., 2015). Second, job crafting reshapes the content and boundaries of work. Employees gain more opportunities to identify new challenges and contradictions in their work environment, which can spark innovative ideas (Wang, 2021). Additionally, job crafting transforms employees’ perceptions of their work, helping them find meaning in their roles (Wrzesniewski et al., 2013) and influencing their innovative performance (Tuan, 2018). Finally, job crafting also involves changing relationships by expanding interpersonal resources and interactions. These resources support employees in promoting new ideas and foster an environment that encourages innovative activities (Afsar et al., 2019). Therefore, we propose the following hypothesis:

*H2*: Job crafting mediates the relationship between digital leadership and employee innovative performance in the form of (2a) task crafting, (2b) cognitive crafting, and (2c) relational crafting.

### 3.3 Moderating role of person–job fit

Person–job fit is an important aspect of person–environment fit (Yu, 2016). Person–job fit refers to the degree of fit between the person and the tasks and goals of the job (Caldwell and O’Reilly, 1990). Person–job fit can be divided into two broad categories: demands–abilities (D–A) fit and needs–supplies (N–S) fit (Edwards, 1991). D–A fit refers to the match between environmental (i.e., job) demands and personal abilities, focusing on whether employees can meet the requirements of their job positions. N–S fit refers to the fit between the needs of a person and the ability of the environment to meet those needs, and focuses on how well the organization meets the needs of its employees and whether, for example, the reward offered by the company matches the contribution of the employee (Cable and DeRue, 2002).

Based on Holland’s (1996) theory of vocational interests, the alignment between an individual’s vocational interests and their work environment is a critical factor influencing work behaviors and performance. The theory suggests that when employee vocational interests align with their work environment, they are more likely to demonstrate positive work attitudes and engage in effective behaviors, ultimately improving performance. Therefore, with a high level of person–job fit, the working styles, skills, and competencies of employees after job crafting will be better aligned with the needs of the organization. When employees feel that their new ways of working, skills, and abilities can be activated in the work environment, they will achieve higher work performance (Lee et al., 2008). Second, a higher person–job fit is conducive to higher job satisfaction and organizational commitment as well as lower employee turnover (Saks and Ashforth, 1997). In such an atmosphere, employees involved in job crafting will gain a strong sense of job security, which will be more conducive to generating innovative ideas and implementing them, thus increasing innovative performance. Finally, a higher person–job fit leads to a better match between the cognitive abilities of the employee and the characteristics of the job itself, thus increasing the likelihood of the employee showing innovative behavior (Huang et al., 2019). Therefore, person–job fit affects the relationship between job crafting and employee innovation performance, resulting in the following hypothesis.

*H3*: Person–job fit positively moderates the relationship between job crafting and employee innovative performance in the form of (3a) task crafting, (3b) cognitive crafting, and (3c) relational crafting, such that the relationship becomes stronger when person–job fit is high rather than low.

### 3.4 Moderated mediating effect

Prior studies have highlighted that person-job fit enhances proactive behavior effectiveness by aligning employees’ competencies with job demands (Cable and DeRue, 2002; Huang et al., 2019). H2 and H3 together form a mediation model with moderating, which is based on the moderating mediator inference method (Edwards and Lambert, 2007). Person–job fit moderates the relationship between job crafting and employee innovative performance. This study further predicted that person–job fit positively moderates the mediating effects of digital leadership on employee creativity via job crafting. Therefore, the following hypothesis is proposed.

*H4*: Person–job fit positively moderates the indirect effect of digital leadership on employee innovative performance through job crafting in the form of (4a) task crafting, (4b) cognitive crafting, and (4c) relational crafting. In other words, the higher the person–job fit, the greater the mediating effect of task crafting, cognitive crafting, and relational crafting.

**FIGURE 1:**
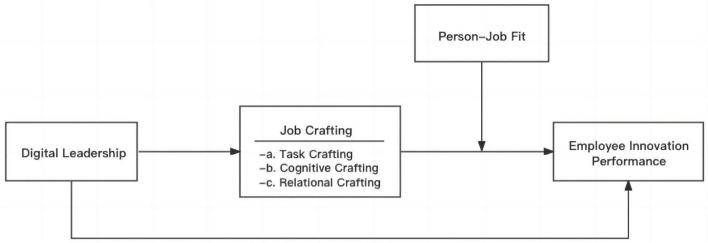
Theoretical model.

Figure 1 shows the theoretical model developed in this study.

## 4 Materials and methods

### 4.1 Sample and procedures

Data for this study were collected from March 2024 to April 2024. The research sample mainly included employees from manufacturing firms that are implementing digital transformation in the Shandong province of China. Among the 20 companies approached a total of 6 entities accorded their consent to participate in the study. A formal survey was conducted using a web-based questionnaire. Approval was guaranteed by the relevant human resource heads of the companies, who willingly participated in the surveys. This study separated the independent variables from the dependent variables in survey waves to mitigate common method bias (Podsakoff et al., 2012). The questionnaire survey comprised two stages: During Time 1 (T1), employees completed questionnaires regarding a predictor variable (digital leadership), a mediating variable (job crafting), and demographic variables (age, gender, education, and seniority). After a month, during Time 2 (T2), the same participants completed questionnaires regarding a moderating variable (person–job fit) and a dependent variable (employee innovative performance). To match the responses obtained during T1 and T2, participants were asked to enter the last four digits of their ID numbers in the questionnaires.

A total of 455 questionnaires were distributed in this study and 370 questionnaires were collected. After filtering out incomplete responses and removing outliers, 306 valid questionnaires remained with a response rate of 67.3% In terms of the sample distribution, 187 (61.11%) respondents were males and 119 (38.89%) were females. Most of the respondents were aged between 26 and 35 years (35.62% of the total survey population). The majority possessed a bachelor’s degree (43.46% of the total survey population) and seniority ranged mostly between 4 and 6 years (27.45% of the total survey population) (Table 1). The sample size was adequate to analyze the model. Using the rule of thumb of (no. of items in questionnaire X 5 = 48 X5) which is 240 (Faul et al., 2009). Therefore, the sample of 306 is adequate because this is more than the required number of 240 responses.

**TABLE 1:** Demographics of the survey respondent.

Variable		*N*	Percentage			*N*	Percentage
Gender	Male	187	61.11	Education	High school	29	9.48
	Female	119	38.89		Associate degree	92	30.07
Age	≤25	44	14.38		Bachelor degree	133	43.46
	[26, 35]	109	35.62		Master degree	46	15.03
	[36, 45]	83	27.12		Doctoral degree	6	1.96
	[46, 55]	59	19.28	Seniority	≤1	26	8.5
	≥56	11	3.60		[1, 3]	44	14.38
					[4, 6]	84	27.45
					[7, 10]	64	20.92
					≥10	88	28.76

### 4.2 Measures

The scales used in this study were mainly derived from mature scales used in the academic community, with proven reliability and validity in domestic and foreign studies. All scales used the 5-point rating like the Likert scale, where 1 means strongly disagree and 5 means strongly agree. The specific measurement of each variable is as follows.

### 4.3 Digital leadership

Digital leadership was measured with the 17-item scale adapted by Wang et al. (2022) based on the measure developed by Zhu (2015), containing five dimensions: creativity, thinking and inquisition, curiosity, deep knowledge, and global vision and collaboration. However, consistent with our contextual theoretical framework, we considered the scale as a single construct measuring organizational empowerment. The items were as follows: “The top leader of our company has the capability to implement the learning and digital capability,” “The top leader of our company has creativity and an innovative mindset,” etc. Cronbach’s alpha for this scale was 0.946.

### 4.4 Job crafting

Job crafting was measured with the 15-item scale developed by Slemp and Vella-Brodrick (2013), containing 3 dimensions: task crafting, cognitive crafting, and relational crafting. The items are as follows: “Introduce new approaches to improve your work,” “Think about how your job gives your life purpose,” and “Make an effort to get to know people well at work.” Cronbach’s alpha values for this scale were 0.860, 0.879, and 0.897, respectively, for the three dimensions.

### 4.5 Employee innovative performance

Employee innovative performance was measured with the 10-item scale developed by Janssen (2000). The items are as follows: “I will provide new ideas to improve the current situation,” “I can transform innovative ideas into reality application” etc. Cronbach’s alpha for this scale was 0.921.

### 4.6 Person–job fit

Person–job fit was measured with the 6-item scale developed by Cable and DeRue (2002), The items are as follows: “There is a good fit between what my job offers me and what I am looking for in a job,” “The attributes that I look for in a job are fulfilled very well in my present job” etc. Cronbach’s alpha for this scale was 0.892.

## 5 Results

All statistical analyses were conducted using SPSS 29.0, Mplus 8.0, and SmartPLS 4.1. SEM was run with Mplus 8.0 and SmartPLS 4.1 to test reliability, validity, and model fit. To examine the hypotheses, this study performed partial least squares structural equation modeling (PLS-SEM) using SmartPLS 4.1 software.

### 5.1 Reliability and validity

To assess the reliability of the constructs, we first calculated Cronbach’s alpha to test the reliability. The Cronbach’s alpha value of each construct ranged from 0.860 to 0.946 (see Table 2), which was greater than the recommended threshold value of 0.70, indicating adequate reliability (Gliem and Gliem, 2003).

**TABLE 2:** Construct reliability and validity.

Items	Loading	Cα	CR	AVE
**Digital leadership**		0.946	0.952	0.538
Item1	0.859			
Item2	0.755			
Item3	0.739			
Item4	0.717			
Item5	0.689			
Item6	0.730			
Item7	0.734			
Item8	0.772			
Item9	0.714			
Item10	0.765			
Item11	0.696			
Item12	0.736			
Item13	0.687			
Item14	0.722			
Item15	0.718			
Item16	0.720			
Item17	0.702			
**Task crafting**		0.860	0.899	0.642
Item1	0.884			
Item2	0.744			
Item3	0.817			
Item4	0.754			
Item5	0.798			
**Cognitive crafting**		0.879	0.912	0.675
Item6	0.886			
Item7	0.821			
Item8	0.800			
Item9	0.772			
Item10	0.823			
**Relational crafting**		0.897	0.924	0.708
Item11	0.905			
Item12	0.848			
Item13	0.802			
Item14	0.796			
Item15	0.850			
**Employee innovative performance**		0.921	0.934	0.585
Item1	0.906			
Item2	0.750			
Item3	0.726			
Item4	0.772			
Item5	0.731			
Item6	0.756			
Item7	0.727			
Item8	0.749			
Item9	0.742			
Item10	0.775			
**Person–job fit**		0.892	0.917	0.650
Item1	0.869			
Item2	0.820			
Item3	0.761			
Item4	0.789			
Item5	0.796			
Item6	0.796			

*N* = 306; Cα, Cronbach’s alpha; CR, composite reliability; AVE, average variance.

Second, CFA was conducted to calculate the overall measurement model’s convergent and discriminant validity. The model fulfilled (Hair et al., 2019) criteria for convergent validity, with factor loadings ranging from 0.687 to 0.906 (all exceeding 0.6; *p* < 0.001). Additionally, CR values ranged from 0.899 to 0.952 (surpassing 0.7), and AVE values ranged from 0.538 to 0.708 (exceeding 0.5). AVE, CR, and Cα-values for each construct are presented in Table 2.

For discriminant validity, compared to other competition models, the theoretical six-factor model (digital leadership, task crafting, cognitive crafting, relational crafting, person–job fit, and employee innovative performance) had a better fit to the data [χ^2^/df = 1.152, (CFI) = 0.980, (TLI) = 0.979, (RMSEA) = 0.022, and (SRMR) = 0.046 (see Table 3). The CFA results showed that the theoretical six-factor model had satisfactory discriminant validity.

**TABLE 3:** Results of confirmatory factor analysis.

Models	Factor	χ^2^	df	χ^2^/df	RMSEA	CFI	TLI	SRMR
Six-factor model	DL, TC, RC, CC, EIP, PJF	1226.353	1065	1.152	0.022	0.980	0.979	0.046
Four-factor model	DL, TC+RC+CC, EIP, PJF	2608.537	1074	2.429	0.068	0.814	0.805	0.079
Three-factor model	DL, TC+RC+CC, EIP+ PJF	3437.981	1077	3.192	0.085	0.714	0.701	0.101
Two-factor model	DL+TC+RC+CC+EIP, PJF	4483.590	1079	4.155	0.102	0.588	0.596	0.114
Single-factor model	DL+TC+RC+CC+EIP+PJF	5176.710	1080	4.793	0.111	0.504	0.482	0.121

*N* = 306; DL, digital leadership; TC, task crafting; CC, cognitive crafting; RC, relational crafting; PJF, Person–Job Fit; EIP, employee innovative performance.

Furthermore, the heterotrait–monotrait ratio of correlations (HTMT) criteria were employed to test the discriminant validity. Different recommendations exist for confirming the HTMT criterion, with the conservative criterion suggesting that the HTMT value should be below 0.85 (Tabri and Elliott, 2012), and the classical criterion indicating that the HTMT value should be below 0.90 (Henseler et al., 2015). The HTMT ratio table demonstrates that all values fall within the range of 0.083-0.554, which is lower than the specified criterion, thus confirming discriminant validity (Table 4).

**TABLE 4:** Heterotrait–monotrait ratio (*n* = 306).

	DL	TC	CC	RC	EIP	PJF
DL						
TC	0.446					
CC	0.475	0.472				
RC	0.178	0.083	0.157			
EIP	0.445	0.463	0.554	0.123		
PJF	0.421	0.403	0.436	0.091	0.358	

### 5.2 Common method variance

Common method variance (CMV) may affect the empirical results because our study data were collected through self-report questionnaires. Podsakoff et al. (2003) showed that procedural and statistical techniques can be adopted for CMV. In the statistical technique, the possibility of common method bias was tested using Harman’s one factor test (Podsakoff and Organ, 1986). A principal component factor analysis with varimax rotation was used on the items of digital leadership, job crafting, P–J fit, and employee innovative performance. This result revealed multiple factors with eigenvalues greater than 1. The first factor accounted for 19.35% ( < 50%) loading, which proved the absence of CMV (Woszczynski and Whitman, 2004). Further, we conducted the unmeasured latent method factor (Podsakoff et al., 2003), to test CMV.

A comparison of the latent method factor model (χ^2^/df = 1.059, CFI = 0.993, TLI = 0.992, RMSEA = 0.014, SRMR = 0.037) and the six-factor model (χ^2^/df = 1.152, CFI = 0.980, TLI = 0.979, RMSEA = 0.022, SRMR = 0.046) indicated no significantly changes in CFI (Cheung and Rensvold, 2002). Thus, CMV was not a major problem for the data (Podsakoff et al., 2003).

### 5.3 Means and correlations

The descriptive statistics and correlation analysis results presented in Table 5 indicate that digital leadership is positively correlated to task crafting (*r* = 0.398**), cognitive crafting (*r* = 0.429**), relational crafting (*r* = 0.161**), and employee innovative performance (*r* = 0.414**). Task crafting and cognitive crafting are positively correlated to employee innovative performance (*r* = 0.412** and *r* = 0.495**). The correlation between the key variables supports our hypotheses on the direct and indirect effects of digital leadership on employee innovative performance.

**TABLE 5:** Means, standard deviations (SD), and correlations.

Variables	Mean	SD	Gender	Age	Education	Seniority	DL	TC	CC	RC	PJF	EIP
Gender	1.39	0.488	1									
Age	2.62	1.062	0.582**	1								
Education	2.70	0.906	0.221**	-0.017	1							
Seniority	3.47	1.276	0.511**	0.897**	0.021	1						
DL	3.35	0.802	0.039	-0.021	0.045	0.003	1					
TC	3.21	0.916	0.046	-0.046	0.002	-0.030	0.398**	1				
CC	3.23	0.945	0.175**	0.079	-0.005	0.099	0.429**	0.405**	1			
RC	3.02	1.039	0.074	0.025	0.054	-0.030	0.161**	0.028	0.137*	1		
PJF	3.23	0.952	-0.051	-0.058	0.022	-0.068	0.385**	0.351**	0.384**	0.079	1	
EIP	3.07	0.836	0.091	0.030	-0.032	0.043	0.414**	0.412**	0.495**	0.099	0.324**	1

*N* = 306; **p < 0.01; *p < 0.05.

### 5.4 Structural model

Before testing the structural model, we first examined the R^2^-value, which indicates the model’s predictive power by showing the endogenous variable’s variance that the exogenous variables can explain. The R^2^-value for EIP (0.337) indicates that all the constructs combined explain 33.7% of the variance in EIP. The R^2^-values for the other variables were TC (0.166), CC (0.193), and RC (0.028). Further, we checked the Q^2^-values to assess the predictive relevance values generated by the variables. The Q^2^-values for EIP (0.171), TC (0.156), CC (0.184), and RC (0.021) were above 0, which means that the model has predictive relevance.

To examine the hypotheses, bootstrapping was carried out using SmartPLS 4.1 with 5000 subsamples based upon percentile bootstrapping with a two-tailed test type and a significance level of 0.05. The PLS-SEM bootstrapping approach statistically determined the structural mode coefficients representing the hypothesized relationships.

### 5.5 Direct effect and mediation effect testing

**FIGURE 2:**
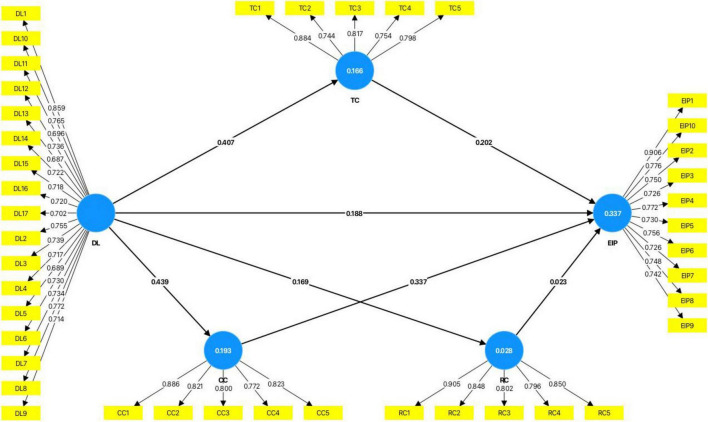
A structural model with mediation effects.

Figure 2 and Table 6 portray the results of the structural path analysis. The results show that digital leadership has a significant positive impact on employee innovative performance (*B* = 0.188; *P* < 0.001; 95% CI: 0.084–0.291), supporting H1. Further, the indirect effect through task crafting (*B* = 0.082; *P* < 0.001; 95% CI: 0.041–0.132) and the indirect effect through cognitive crafting (*B* = 0.148; *P* < 0.001; 95% CI: 0.100–0.205) are significant. The indirect effect through relational crafting is insignificant (*B* = 0.004; *P* = 0.670; 95% CI: -0.014–0.024). Therefore, H2a and H2b are supported, whereas H2c is not.

**TABLE 6:** Results of main and mediation effect.

Hypotheses	Relationship	*B*	SD	T statistics	*P*-value	LLCI	ULCI	Results
	DL - > TC	0.407	0.045	8.964	0.000	0.320	0.499	Supported
	DL - > CC	0.439	0.045	9.820	0.000	0.351	0.530	Supported
	DL - > RC	0.169	0.049	3.464	0.001	0.085	0.276	Supported
	TC - > EIP	0.202	0.052	3.916	0.000	0.099	0.304	Supported
	CC - > EIP	0.337	0.051	6.618	0.000	0.235	0.438	Supported
	RC - > EIP	0.023	0.050	0.461	0.645	-0.075	0.124	Not supported
H1	DL - > EIP	0.188	0.053	3.552	0.000	0.084	0.291	Supported
H2a	DL - > TC - > EIP	0.082	0.023	3.612	0.000	0.041	0.132	Supported
H2b	DL - > CC - > EIP	0.148	0.027	5.541	0.000	0.100	0.205	Supported
H2c	DL - > RC - > EIP	0.004	0.009	0.427	0.670	-0.014	0.024	Not supported

### 5.6 Moderating effect testing

**FIGURE 3:**
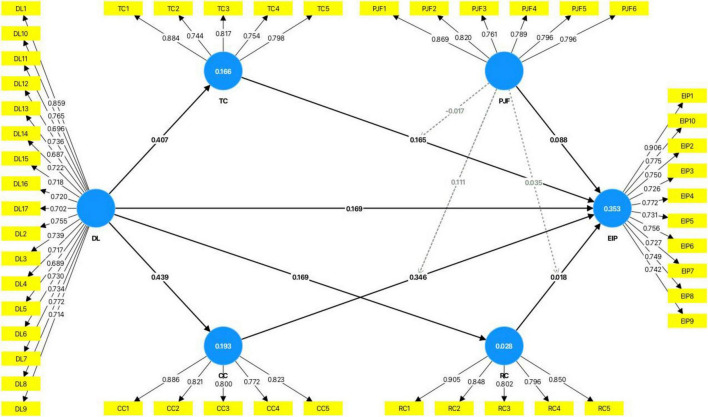
A structural model with moderating effects.

**FIGURE 4:**
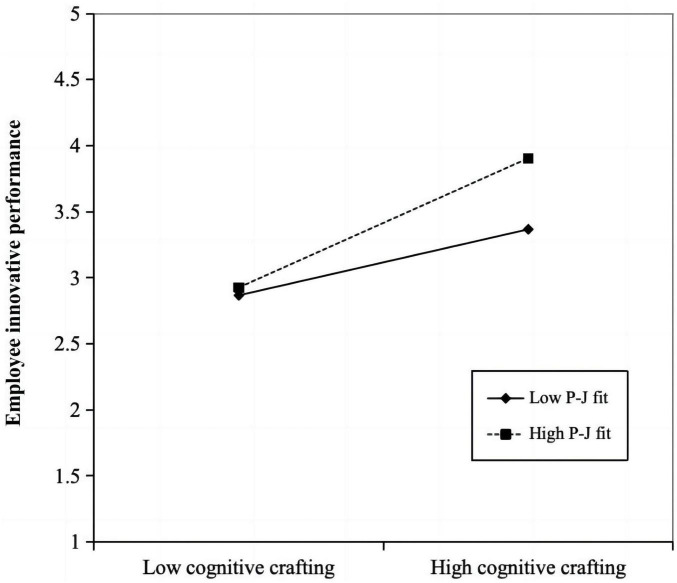
Moderating effect of person–job fit on the relationship between cognitive crafting and employee innovative performance.

We examined the moderating effect of person–job fit on the relationship between job crafting (task crafting, cognitive crafting, and relational crafting) and employee innovative performance. As shown in Figure 3 and Table 7, the interaction between cognitive crafting and person–job fit is significantly and positively related to employee innovative performance (*B* = 0.111; *P* < 0.05; 95% CI: 0.018–0.208), indicating that person–job fit positively moderates the relationship between cognitive crafting and employee innovative performance. Hence, H3b is supported. The interaction between task redesign, relational crafting, and person–job fit is not significantly related to employee innovative performance (*B* = -0.017; *P* = 0.727; 95% CI: -0.112–0.084, *B* = 0.035; *P* = 0.496; 95% CI: -0.070–0.134). Hence, H3a and H3c are not supported. According to the suggestions of Toothaker (1994), this study further analyzed the moderating effect by testing the simple slopes at high and low levels of person–job fit, and the moderating effect diagram was drawn (see Figure 4).

**TABLE 7:** Results of moderating effect.

Hypotheses	Relationship	B	SD	T statistics	*P*-value	LLCI	ULCI	Results
H3a	PJF × TC - > EIP	-0.017	0.049	0.349	0.727	-0.112	0.084	Not supported
H3b	PJF × CC - > EIP	0.111	0.048	2.302	0.021	0.018	0.208	Supported
H3c	PJF × RC - > EIP	0.035	0.052	0.681	0.496	-0.070	0.134	Not supported

### 5.7 Moderated mediation testing

As it was concluded that H3a and H3c were not valid when evaluating the moderating effect, H4a and H4c were directly rejected and only H4b was tested. The index of moderated mediation (Hayes, 2015) presented in Table 8 indicates the total moderated mediation effect. The effect was significant (*B* = 0.062; *P* < 0.01; 95% CI: 0.026–0.102), indicating that the indirect effect of digital leadership on employee innovative performance through cognitive crafting was moderated by person–job fit.

**TABLE 8:** Results of moderated mediation effect.

	Knowledge sharing	*B*	SD	T statistics	*P*-value	LLCI	ULCI
DL - > CC - > EIP	High (+1SD)	0.229	0.039	5.862	0.000	0.157	0.310
	Middle	0.171	0.030	5.714	0.000	0.115	0.232
	Low (-1SD)	0.112	0.035	3.199	0.001	0.044	0.181
Index of conditional mediation		0.062	0.023	2.669	0.008	0.026	0.102

The conditional indirect effect on the values of the moderator was calculated, namely the mean, one standard deviation above, and one standard deviation below. The results are shown in Table 8. The model of digital leadership influencing employee innovative performance via cognitive crafting shows that at low levels of person–job fit, the mediating effect of cognitive crafting is significant (*B* = 0.112; *P* < 0.01; 95% CI: 0.044–0.181). At high levels of person–job fit; the mediating effect of employee cognitive crafting is significant (*B* = 0.229; *P* < 0.001; 95% CI: 0.157–0.310). The analysis results show that the higher the person–job fit, the stronger the mediating role of cognitive crafting in the relationship between digital leadership and employee innovative performance, thus supporting H4b.

## 6 Discussion

Building on the proactive motivation model, this study examined the relationship between digital leadership and employee innovative performance with job crafting as the mediator and person–job fit as the moderator. The research hypotheses were evaluated theoretically and empirically using SPSS, Mplus, and SmartPLS, yielding various interesting findings.

First, digital leadership is positively related to employee innovative performance. The higher the level of digital leadership shown by a leader, the more effective it is in stimulating employee innovative performance. Digital leaders can improve employees’ innovative performance by cultivating a digital culture, allocating essential resources, and streamlining organizational processes. These findings agree with the results of Mihardjo et al. (2019) and Zhu et al. (2022). Thus, this study offers novel insights into the study of digital leadership.

Second, the results of this study show that task crafting and cognitive crafting mediate the relationship between digital leadership and employee innovation performance. Job crafting is an effective way for employees to cope with the digital economic environment. Digital technology has changed the traditional way of working (Gilson et al., 2015). Therefore, under the guidance of digital leadership, employees may proactively adjust task content and optimize task processes to better align with their skills and interests. Additionally, they may redefine the meaning of their work, perceiving it as a creative endeavor. This process enhances employees’ identification with their work, fosters intrinsic motivation, and ultimately facilitates innovative performance (Zhang and Parker, 2019). However, the mediating effect of relational crafting was not significant, probably because the variable ‘relationship’ cannot be absolutely quantified. Furthermore, the perceived degree of relational crafting varied between individuals. The potential for opposing effects could lead to a weakening of the effectiveness of relational crafting.

Third, person–job fit positively moderates the relationship between cognitive crafting and employee innovative performance, as well as the indirect effect of digital leadership on employee innovative performance through cognitive crafting. Cognitive crafting involves employees redefining the meaning of their work and their self-identity (Wrzesniewski and Dutton, 2001), and a high person–job fit implies that the work style, skills, and competencies of an employee are highly compatible with the needs of the organization (Cable and DeRue, 2002). When employees undergo cognitive crafting in environments with a high person–job fit, they are more likely to experience a high degree of congruence between their work and personal perceptions, which in turn leads to greater motivation and promotes innovative performance. However, person–job fit does not moderate the influence of the relationship between task crafting and relational crafting on employee innovation performance. This may be because of the following reasons: (1) Task crafting and relational crafting involve adjustments to work tasks and personal social relationships. These two forms of crafting are more dependent on the organizational environment and structure as well as external conditions of job roles, which may limit the moderating effect of person–job fit. (2) Cognitive crafting has higher autonomy, with lower implementation costs and risks. In contrast, task crafting and relational crafting face greater complexity, uncertainty, and resource demands in practice.

### 6.1 Theoretical contributions

First, this study examined the relationship between digital leadership and employee innovative performance. Existing literature shows that the impact of digital leadership on employees is mainly in terms of performance (Turyadi et al., 2023), capability (Retnowati and Santosa, 2023), and creativity (Zhu et al., 2022). This study is a step toward filling this gap by exploring the relationship between digital leadership and employee innovative performance. Results show that digital leadership does improve employee innovative performance, thus enriching the literature on the antecedents of employee innovative performance.

Second, this study is grounded in the Proactive Motivation Model (Parker et al., 2010) and develops a mediation model linking digital leadership, job crafting, and innovative performance from a role motivation perspective. This model explains how digital leadership influences employee innovative performance through job crafting and highlights the unique motivational mechanisms provided by digital leadership. Few studies have focused on the mechanisms by which digital leadership mediates job crafting. Based on the foundational research of Wrzesniewski and Dutton (2001), this study categorized job crafting into three dimensions and examined the mediating role of each of these dimensions in the relationship between digital leadership and employee innovation performance. Results show that task crafting and cognitive crafting mediate the relationship between digital leadership and employee innovative performance, but relational crafting does not show the same effect. This finding provides a more nuanced view of job crafting, revealing the differential impact of different types of job crafting on employee innovation performance.

Third, this study used person–job fit as a moderating variable in the relationship between job crafting and employee innovation performance. A high person–job fit enhanced the positive impact of cognitive crafting on innovation performance but had no significant effect on the outcomes of task crafting and relational crafting. Further, the results highlight the important role of person–job fit in organizational behavior, suggesting that person–job fit may contribute to the positive impact of cognitive crafting on innovation performance by enhancing the intrinsic motivation of employees. This study also revealed that person–job fit moderates the mediating role of cognitive crafting in the relationship between digital leadership and employee innovative performance. It deepens the understanding of the boundaries of digital leadership affecting employee innovative performance through cognitive crafting.

### 6.2 Practical implications

First, in the digital economy, employee innovation is a key enabler of business growth. Companies and managers should recognize the unique ability of digital leadership to foster innovation. Managers can strive to improve their digital literacy and skills in various ways, such as deliberate learning and active participation in relevant trainings and forums.

Second, the findings suggest that task crafting and cognitive crafting mediate the relationship between digital leadership and employee innovation performance. Overall, although job crafting is an employee-initiated activity, it can be influenced to some extent by managers. Specifically, managers can enhance the positive impact of digital leadership on employees by focusing on the task and cognitive aspects of job crafting and providing opportunities for employees to engage in these activities. Therefore, managers should prioritize the creation of flexible work environments that allow for task autonomy and support and encourage employees to cognitively redefine their work roles. For example, managers should encourage employees to set personal development goals and regularly seek their feedback on work-related matters. This alignment of work tasks, personal values, and career aspirations can in turn enhance employee innovation performance.

Third, the results indicate that person–job fit positively moderates the mediating effect of cognitive crafting in the relationship between digital leadership and employee innovation performance. A high person–job fit facilitates full exploitation of the benefits of cognitive crafting. Therefore, organizations should provide ongoing training and development opportunities to help employees improve their skills and maintain high levels of alignment with their evolving job roles. In addition, reward mechanisms encouraging employees to propose innovative solutions during the cognitive crafting process are essential. Involving employees in practical projects can enable them to apply the results of cognitive crafting and test their innovative ideas in real-life scenarios.

### 6.3 Limitations and future research

First, this study collected data at different time points to mitigate the issue of common method variance (CMV) and to somewhat capture the causal relationships between variables over time. However, as all variables were self-reported by employees, CMV could still be a concern. Future research should employ multi item point and multisource data collection methods to address this issue more robustly. Additionally, more rigorous experimental designs, such as matched-pair studies, longitudinal designs, and experimental methods, should be considered to strengthen the validity of the findings.

Second, this study confirms that task crafting and cognitive crafting mediate the relationship between digital leadership and employee innovation performance at the individual level. However, the study does not provide a thorough explanation for the non-significant mediating effect of relational crafting. This result may be attributed to the characteristics of the study sample, as manufacturing firms often feature highly centralized, multi-tiered structures. In these organizations, communication channels and collaboration rules are tightly controlled. While digital leadership facilitates cross-departmental collaboration, organizational structural constraints may restrict employee autonomy in relational crafting, potentially diminishing or even negating its impact on innovative performance. Future research could compare various organizational types or hierarchical structures and apply multilevel analysis to explore the varying effects of employees’ relational crafting on innovative performance.

Third, this study considers the moderating effect of person–job fit on the relationship between cognitive crafting and employee innovative performance, but it did not confirm its moderating effect on the relationship between task crafting and relational crafting with respect to employee innovative performance. However, person–job fit is only one of the boundary conditions for determining how job crafting affects innovative performance. In reality, many other factors may influence this process. Future research should explore the boundary conditions that influence the impact of job crafting on innovative performance from different perspectives to gain a more comprehensive understanding of this relationship.

Fourth, although this study used Janssen (2000) Innovative Work Behavior (IWB) scale, which was designed to capture key behaviors in the innovation process (e.g., idea generation, promotion, and implementation), the scale primarily emphasizes the process dimension of innovative behavior, rather than directly assessing innovation outcomes. While this measurement choice aligns with much of the literature (Anderson et al., 2014), it may not fully capture the conversion of innovative behavior into ultimate performance outcomes. Future research could integrate multi-source data (e.g., number of innovation projects, patent outputs, qualitative evaluations from supervisors or peers) or adopt longitudinal designs to track the lagged effects between behavior and outcomes, thereby systematically revealing the dynamic relationship between innovative behavior and performance.

## Data Availability

The raw data supporting the conclusions of this article will be made available by the authors, without undue reservation.
